# Public Knowledge, Attitudes, and Practices Behaviors Towards Coronavirus Disease 2019 (COVID-19) During a National Epidemic—China

**DOI:** 10.3389/fpubh.2021.638430

**Published:** 2021-03-19

**Authors:** Yuan Xu, Guofu Lin, Claudio Spada, Huifen Zhao, Shuo Wang, Xiaoyang Chen, Yunfeng Chen, Yixiang Zhang, Giuseppe A. Marraro, Xiaohong Zeng, Xiangjia Ye, Li Zhang, Yiming Zeng

**Affiliations:** ^1^Department of Respiratory Pulmonary and Critical Care Medicine, The Second Affiliated Hospital of Fujian Medical University, Quanzhou, China; ^2^Respiratory Medicine Center of Fujian Province, Quanzhou, China; ^3^The Second Clinical College, Fujian Medical University, Quanzhou, China; ^4^Healthcare Accountability Lab, University of Milan, Milan, Italy; ^5^Department of Nursing Teaching and Research, The Second Affiliated Hospital of Fujian Medical University, Quanzhou, China; ^6^The School of Nursing, Fujian Medical University, Fuzhou, China; ^7^Department of Preventive Medicine, The Second Affiliated Hospital of Fujian Medical University, Quanzhou, China

**Keywords:** COVID-19, KAP, knowledge, attitude, practice, survey, China

## Abstract

**Background:** The rapid outbreak of coronavirus disease 2019 (COVID-19) posed a serious threat to China, followed by compulsive measures taken against the national emergency to control its further spread. This study was designed to describe residents' knowledge, attitudes, and practice behaviors (KAP) during the outbreak of COVID-19.

**Methods:** An anonymous online questionnaire was randomly administrated to residents in mainland China between Mar 7 and Mar 16, 2020. Residents' responses to KAP were quantified by descriptive and stratified analyses. A Multiple Logistic Regression model was employed to identify risk factors associated with KAP scores.

**Results:** A total of 10,195 participants were enrolled from 32 provinces of China. Participants of the ≥61 years group had higher KAP scores [adjusted Odds Ratio (ORadj) = 4.8, 95% Confidence Interval (CI): 3.0–7.7, *P* < 0.0001], and the married participants and those in low-income families had higher scores of KAP (ORadj = 1.2, 95% CI: 1.1–1.3; ORadj = 1.8, 95% CI: 1.6–2.2, respectively, both *P* < 0.0001). The participants living with more than two family members had higher scores in an increasing ORs when the family members increased (ORadj = 1.3, 95% CI: 1.1–1.6, *P* = 0.013; ORadj = 1.3, 95% CI: 1.1–1.6, *P* = 0.003; ORadj = 1.3, 95% CI: 1.0–1.6, *P* = 0.02; for groups of 2, 3–4 and ≥5, respectively).

**Conclusions:** Out of the enrolled participants who completed the survey, 85.5% responded positively toward the mandatory public health interventions implemented nationwide by the Chinese authorities. These effective practices seem to be related to a proper attitude generated by the increased knowledge and better awareness of the risks related to the COVID-19 pandemic and the consequent need for safe and responsible behavior.

## Introduction

The Coronavirus Disease 2019 (COVID-19) has experienced an outbreak across China and other countries around the world widely involving the population and the authorities (1). Due to the rapid person-to-person transmission and the asymptomatic initial appearance, with a median incubation period of ~ 5 days, COVID-19 has created a public health emergency of international concern. At the time we conducted our survey, ~1,300,000 confirmed COVID-19 cases had been reported overall the world, including 80,000 deaths across more than 200 countries. Although the number of confirmed cases is still soaring around the world, China has controlled the spread of epidemic. As reported by the National Health Commission of the People's Republic of China, at the moment of preparation of this manuscript, the confirmed cases were 8,976 and the cumulative deaths were 3,226, exhibiting a striking decreasing trend (2).

The outbreak of COVID-19 creates a huge disaster to China, especially during the Chinese Annual Lunar New Year time when the people celebrate in grand pomp and the community participates for several days. Due to the spread through respiratory droplets, the initial epidemic and subsequent pandemic created an overwhelming burden on the public health emergency management system. To control the diffusion of the infection across the nation, Chinese authorities took measures and preventions to block the transmission among close contacts. Due to the lack of effective vaccines, Chinese authorities focused mainly on the strategies of public health outbreak response as community containment, quarantine, and public education (3, 4). Many gatherings were canceled and prohibited, including congresses, public events, holiday parties, etc. and traffic travel in Wuhan and cities across Hubei province was completely blocked.

In addition, education on COVID-19 was delivered to the public through various media: television, internet, and telephone. Therefore, it not only required the authorities to promptly and effectively respond to the emergency during the holiday travel time, but also required the relevant knowledge of COVID-19 be extensively absorbed by the public. Although the above measures had been successfully used in past epidemics (5, 6), it was the first time that they were administrated extensively across the whole nation. The aim was to increase the awareness of the population on the severity of the disease, reflect on the severity and need for following specific guidelines, and behave toward the pandemic in a way that would block the transmission of COVID-19.

During the outbreak of COVID-19, a nation-wide survey in China to disclose residents' knowledge, attitudes, and practices (KAPs) toward the epidemic was conducted, in order to reveal their perceptions of the risk factors, cognition, and health priorities. The aim of the study was to assess the determinants of knowledge and attitudes toward COVID-19, the practice behaviors of prevention among residents, and to disclose public attitudes toward Chinese authorities and government. Up to now, no KAP study regarding COVID-19 has been conducted in China, and this study addresses that gap. Moreover, it can present important suggestions for the authorities of other countries for what should be done to block the pandemic diffusion and the possible measures to be applied.

## Methods

### Setting and Population

During the outbreak of COVID-19, an online self-administrated questionnaire was administrated randomly to residents of 32 provinces of China between March 7th and March 16th, 2020. An electronic questionnaire was distributed to the mobile phones of residents simultaneously with no stratification conducted for sampling. The survey was anonymous and without any possibility of identification.

The study was conducted according to the principles of Helsinki declaration. The bioethical committees at Fujian Medical University 2nd Affiliated Hospital, China, gave written approval for the study (2020-206).

### Survey Measures

The questionnaire was optimized involving expert Chinese researchers and respiratory doctors with extensive experience in the field for designing and developing questions.

Details of the KAP questionnaire are presented in the Supplementary Tables 1–3, consisting in single-choice questions, multiple-choice questions, and open-ended questions. The questionnaire included four sections: Socio-demographics, Knowledge, Attitudes and Practice Behaviors of the participants. The first section focused on personal basic information, including gender, age, educational status, occupation, marital status, inhabiting status, family income and current direct or indirect involvement with COVID-19 illness. The second section consisting of eight questions regards the knowledge of the incubation period, clinical symptoms, measures of transmission, and preventions of COVID-19. In the third section, the attitudes toward COVID-19 were analyzed through ten questions. Participants who were aware of the risk of infection and practiced healthy behaviors were considered as having a positive attitude toward the epidemic. On the contrary, participants who could not or did not recognize the risk of the infection and the importance of personal protection were considered to be negative. The last section included ten questions to evaluate the practical behaviors of participants during the epidemic of COVID-19.

The knowledge, attitude, and practice measured responses of each question were analyzed by a panel of experts, and the cumulative and respective scores were calculated. A higher score indicated a more positive sensitivity toward COVID-19.

There were two open-ended questions eliciting additional comments to describe how respondents were affected by COVID, and the measures they used to keep their mood comfortable during the epidemic. The responses from the open-ended questions will be analyzed in a further study.

### Statistical Analysis

An exploratory factor analysis was used to reveal the validity and factor structure of the knowledge, attitude, and practice items using principal axis factoring and varimax rotation. Descriptive statistics, including frequencies, percentages, means, and SD, were used to quantify the survey responses. The differences of KAP scores between subgroups of socio-demographic characteristics were compared by ANOVA or Games-Howell test. Univariate and multivariate Logistic regression models were constructed to disclose the associations between the groups of KAP scores and subgroups of socio-demographic characteristics. Considering the skewed distribution, we used the median of scores as a cutoff to divide the KAP scores into the lower scores group and higher scores group. The variables adjusted in the multivariate regression models included: gender, education status, marital status, occupation, family members living together, family income, current status affected by COVID-19 and the appearance of clinical symptoms in the previous 14 days.

Based on the data, the classification and regression tree (CART) methodology models were developed to predict visual scores of KAP (7). Data analysis was completed using SPSS (version 22), python (version 3.8.0), and SAS software (9.2, Cary, NC). Figures in the study were constructed using Apache ECharts open-source library (8). All the tests were two-tailed, and values of *P* < 0.01 were considered statistically significant.

## Results

### Socio-Demographic

A total of 10,195 participants of 32 provinces of China were enrolled through the network, with a response rate of 64.4%. The socio-characteristics of participants were described in the Table 1. The ages of participants to the survey ranged from 10 to 80 years old, with the average of 30.2 ± 8.5 years old. The majority of respondents are identified as female (55.4%), aged 21–40 years (80.7%), college/university educational status (59.5%), married (57.3%), living with 3–4 family members (51.3%), and lower family income (40.3%). The types of occupations were defined by the Chinese standard and the employees of commercial/service industry accounted for the largest proportion, 32.9%. 92.1% of participants stated having not, or probably not, been infected by COVID-19. The majority of participants (87.8%) did not have any clinical symptoms before 14 days before the survey.

**Table 1:** The distribution of participants stratified by socio-demographic characteristics, and the stratified analysis by Univariate and multivariate logistic regression model.

		**Univariate logistic regression**		**Multivariate logistic regression**	
	**No. (%; 95% CI)**	**OR (95% CI)**	***P*-value**	**OR (95% CI)**	***P*-value**
**Gender**					
Male	4,557 (44.6; 43.7–45.6)	1.00		1.00	
Female	5,638 (55.4; 54.4–56.3)	1.02 (0.94–1.10)	0.705	1.09 (1.00–1.18)	0.058
**Age, years**					
≤ 20	1,079 (10.6; 10.0–11.2)	1.00		1.00	
21–40	8,224 (80.7; 80.0–81.4)	2.88 (2.51–3.29)	<0.0001	2.09 (1.80–2.43)	<0.0001
41–60	804 (7.9; 7.4–8.5)	2.49 (2.06–3.00)	<0.0001	2.21 (1.79–2.72)	<0.0001
≥61	88 (0.9; 0.7–1.0)	3.62 (2.31–5.68)	<0.0001	4.78 (2.96–7.72)	<0.0001
**Educational status**					
No formal education/Primary	144 (1.4; 1.2–1.6)	1.00		1.00	
Junior	892 (8.7; 8.2–9.3)	1.58 (1.07–2.31)	0.020	1.30 (0.86–1.95)	0.213
Senior	2,758 (27.1; 26.1–27.9)	2.10 (1.46–3.03)	<0.0001	1.72 (1.16–2.55)	0.007
College/University	6,063 (59.5; 58.5–60.4)	3.59 (2.50–5.16)	<0.0001	2.58 (1.74–3.82)	<0.0001
Graduate or above	338 (3.3; 3.0–3.6)	4.47 (2.93–6.82)	<0.0001	3.27 (2.07–5.15)	<0.0001
**Marital status**					
Single	4,130 (40.5; 39.5–41.5)	1.00		1.00	
Married	5,839 (57.3; 56.2–58.2)	1.39 (1.29–1.51)	<0.0001	1.18 (1.08–1.29)	<0.001
Divorced	191 (1.9; 1.6–2.1)	0.67 (0.50–0.90)	0.008	0.80 (0.58–1.10)	0.169
Widowed	35 (0.3; 0.2–0.5)	0.35 (0.16–0.75)	0.007	0.43 (0.19–0.97)	0.042
**Occupations**					
Managers of government/enterprise	1,095 (10.7; 10.1–11.3)	1.00		1.00	
Professionals	1,448 (14.2; 13.5–14.9)	1.46 (1.24–1.71)	<0.0001	1.46 (1.24–1.73)	<0.0001
Clerks	948 (9.3; 8.7–9.9)	0.99 (0.83–1.18)	0.937	0.95 (0.79–1.14)	0.595
Employees of commercial/service industry	3,361 (33.0; 32.1–33.9)	0.99 (0.87–1.14)	0.944	1.12 (0.97–1.30)	0.121
Workers in agriculture/forestry/animal husbandry/fishing/	448 (4.4; 4.0–4.8)	0.75 (0.60–0.93)	0.009	0.98 (0.78–1.24)	0.872
water conservancy					
Operators of production/transportation equipment	555 (5.4; 5.0–5.9)	1.49 (1.21–1.84)	<0.0001	1.54 (1.23–1.92)	<0.001
Polices/Militaries/Guards	47 (0.5; 0.3–0.6)	0.28 (0.14–0.54)	<0.0001	0.44 (0.21–0.88)	0.020
Others	1,102 (10.8; 10.2–11.4)	0.73 (0.62–0.87)	<0.0001	0.86 (0.72–1.03)	0.103
Unemployed	1,191 (11.7; 11.1–12.3)	0.54 (0.46–0.64)	<0.0001	0.82 (0.68–0.98)	0.033
**Family members living together (No.)**					
Single	596 (5.8; 5.4–6.3)	1.00		1.00	
1	378 (3.7; 3.3–4.1)	0.97 (0.75–1.26)	0.843	0.94 (0.71–1.23)	0.643
2	2,166 (21.2; 20.5–22.0)	1.50 (1.25–1.80)	<0.0001	1.28 (1.05–1.56)	0.013
3–4	5,226 (51.3; 50.2–52.2)	1.58 (1.33–1.87)	<0.0001	1.33 (1.10–1.60)	0.003
≥5	1,829 (17.9; 17.2–18.7)	1.48 (1.23–1.79)	<0.0001	1.27 (1.03–1.55)	0.023
**Family incomes (rmb per year)**					
<50,000	2,935 (28.8; 27.9–29.7)	1.00		1.00	
50,000–120,000	4,112 (40.3; 39.3–41.2)	1.77 (1.62–1.96)	<0.0001	1.43 (1.29–1.58)	<0.0001
130,000–170,000	1,683 (16.5; 15.8–17.2)	2.16 (1.91–2.44)	<0.0001	1.72 (1.50–1.96)	<0.0001
180,000–250,000	930 (9.1; 8.6–9.7)	2.36 (2.03–2.75)	<0.0001	1.82 (1.55–2.15)	<0.0001
>250,000	535 (5.2; 4.8–5.7)	1.77 (1.47–2.13)	<0.0001	1.40 (1.14–1.70)	0.001
**Current status affected by COVID-19**					
Diagnosed, and cured	40 (0.4; 0.3–0.5)	1.00		1.00	
Diagnosed, and under treatment	46 (0.5; 0.3–0.6)	0.32 (0.06–1.74)	0.185	0.25 (0.05–1.39)	0.113
Suspected, and quarantined	93 (0.9; 0.7–1.1)	2.17 (0.76–6.21)	0.149	1.67 (0.57–4.89)	0.347
Home-based quarantine	218 (2.1; 1.9–2.4)	3.60 (1.36–9.57)	0.010	2.67 (0.98–7.22)	0.054
Confirmed healthy after quarantine	410 (4.0; 3.6–4.4)	11.40 (4.37–29.71)	<0.0001	8.26 (3.11–21.93)	<0.001
None of above	9,388 (92.1; 91.5–92.6)	8.51 (3.33–21.73)	<0.0001	5.49 (2.10–14.31)	0.0001
Appearance of clinical symptoms in previous 14 days^*^					
No	8,952 (87.8; 87.2–88.5)	1.00		1.00	
Yes	1,243 (12.2; 11.5–12.8)	0.60 (0.53–0.68)	<0.0001	0.67 (0.58–0.77)	<0.0001

* *Referred to symptoms of fever, cough, expectoration, diarrhea, weak, headache, runny nose, rhinobyon, sore throat*.

### Knowledges, Attitudes, and Practice Behaviors

The questions regarding the knowledge yielded a higher perception on COVID-19 (Supplementary Table 1). Approximately more than 70% reported correct perception of the transmission routes of COVID-19, and more than 88% reported clearly defined terms of “close contact.” 96.4% [95% Confidence Interval (CI) = 96.0–96.8%] of participants reported having perceptions about the typical clinical symptoms of COVID-19, and 52.6% (95% CI = 51.7–53.6%) reported having the conception of its incubation period. 82.0% of responders had the correct perceptions of the measures to be taken when in close contact with confirmed cases. When fever was identified, 1.5% (95% CI = 1.2–1.7%) of respondents had awareness of wearing a mask before diagnosis was confirmed. Ninety percentage reported having the conception of preventive measures implemented by the government. Only 34.5% (95% CI = 33.6–35.5%) reported they would “visit doctors frequently” to prevent COVID-19.

Among the attitudes related to disclosure, nine questions were listed in this section (Supplementary Table 2). The question regarding whether COVID-19 had a serious influence on personal life yielded a “agree” and “strongly agree” response among 47.7% (95% CI = 46.8–48.6%) and 31.9% (95% CI = 31.0–32.8) of respondents, respectively. More than 70% of participants self-rated their worrying about COVID as “a little” (51.2%, 95% CI = 50.3–52.2%) and “very worried” (19.2%, 95% CI = 18.4–20.0%). The question asking whether they were more nervous than ever after having a fever or cough yielded an “agree” and “strongly agree” response among 52.6% (95% CI = 51.6–53.5%) and 20.7% (95% CI = 19.9–21.5%) of respondents, respectively. 43.1% (95% CI = 42.1–43.9%) reported having more concern on the outbreak of COVID-19, and 49.5% (95% CI = 48.6–50.7%) reported being very concerned and being familiar with daily national epidemic trends. In addition, the affected aspects of life primarily focused on transport, working, and shopping, accounting for 87.0, 77.6, 69.5% of respondents, respectively.

Further, the question regarding the satisfaction of the control measures imposed by the government yielded an “agree” and “strongly agree” response of 45.8% (95% CI = 44.8–46.8%) and 40.2% (95% CI = 39.2–41.1%), respectively. The question asking whether individuals had faith in these control measures yielded a “have a strong confidence” and “have confidence” response among 68.4 and 25.3% of respondents, respectively. And the participants self-rated their worrying of COVID-19 as “strongly support” and “support” for the protective measures taken by the government yielded a 76.3% (95% CI = 75.4–77.1%) and 21.8% (95% CI = 21.0–22.6%) of respondents, respectively. Compared with younger people, participants in the ≥61 age group had higher scores on the three questions (*P* < 0.01) (Supplementary Table 4). Participants with college/university educational level were subject to have higher scores of these than those with lower educational levels (*P* < 0.001). The married had higher scores of these than other groups (*P* < 0.001). And the participants with family income ranging from 130,000 to 250,000 also had higher scores on these questions (*P* < 0.05). From the questions mentioned above, we divided the participants into two groups according to the median scores of KAP.

Additionally, nine questions of practice behaviors were listed (Supplementary Table 3). The vast majority of respondents (91.3%, 95% CI = 90.8–91.8%) chose to stay at home during the Lunar New Year holidays, instead of gathering and celebrating outside. 85.5% (95% CI = 84.7–86.2%) chose not to go out even though they were invited by friends. In cases when it was necessary, 87.9% (95% CI = 87.2–88.5%) reported that they kept one-meter distance from each other, and 94.7% (95% CI = 94.3–95.1%) reported that they used a mask for personal protection. However, only 63.1% (95% CI = 62.2–64.0%) of respondents were able to identify that the most correct measures to deal with a used disposable mask was to dispose it into designed dustbin of the community. Secondly, the majority of participants, accounting for 93.0% (95% CI = 92.5–93.5%), made social contact through networks. 5.1% (95% CI = 4.7–5.5%) met friends face to face. Of the total participants, 92.4% (95% CI = 91.9–93.0%) reported usually opening windows for ventilation, and 93.1% (95% CI = 92.6–93.5%) reported choosing household quarantine, and 94.2% (95% CI = 93.7–94.6%) reported wearing a face mask while going out, while 10.1% (95% CI = 9.4–10.7%) reported not taking any protective measures.

For personal daily life, 59.4% (95% CI = 58.4–60.3%) reported shopping for daily necessities by ordering online, and 36.3% (95% CI = 35.4–37.3%) reported doing it under the assistance of community volunteers. 47.0% (95% CI = 46.0–48.9%) reported going shopping by themselves at a market or supermarket. People during the epidemic moved on foot and in private cars, accounting for 57.5% (95% CI = 56.6–58.4%) and 55.6% (95% CI = 54.6–56.5%) of participants, respectively. Participants used public vehicles, such as taxis (9.7%, 95% CI: 9.1–10.2%), buses, and subways (11.5%, 95% CI: 10.8–12.1%).

### Scores on KAP

**Figure 1:**
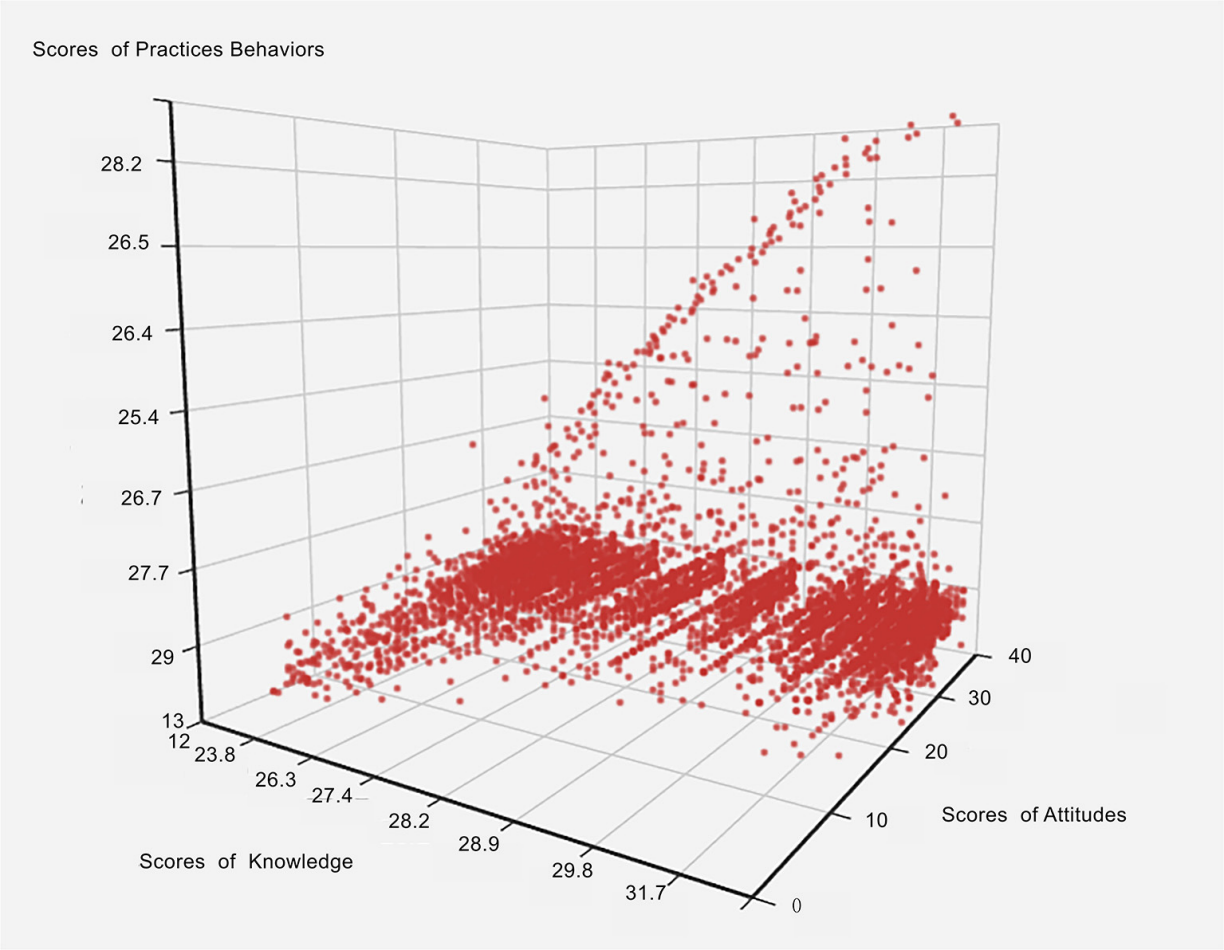
The distributions of scores on knowledge, attitude, and practice manifested by 3D scatter. X axis represents the scores of knowledge, Y axis represents the scores of attitudes, and Z axis represents the scores of practices.

**Figure 2:**
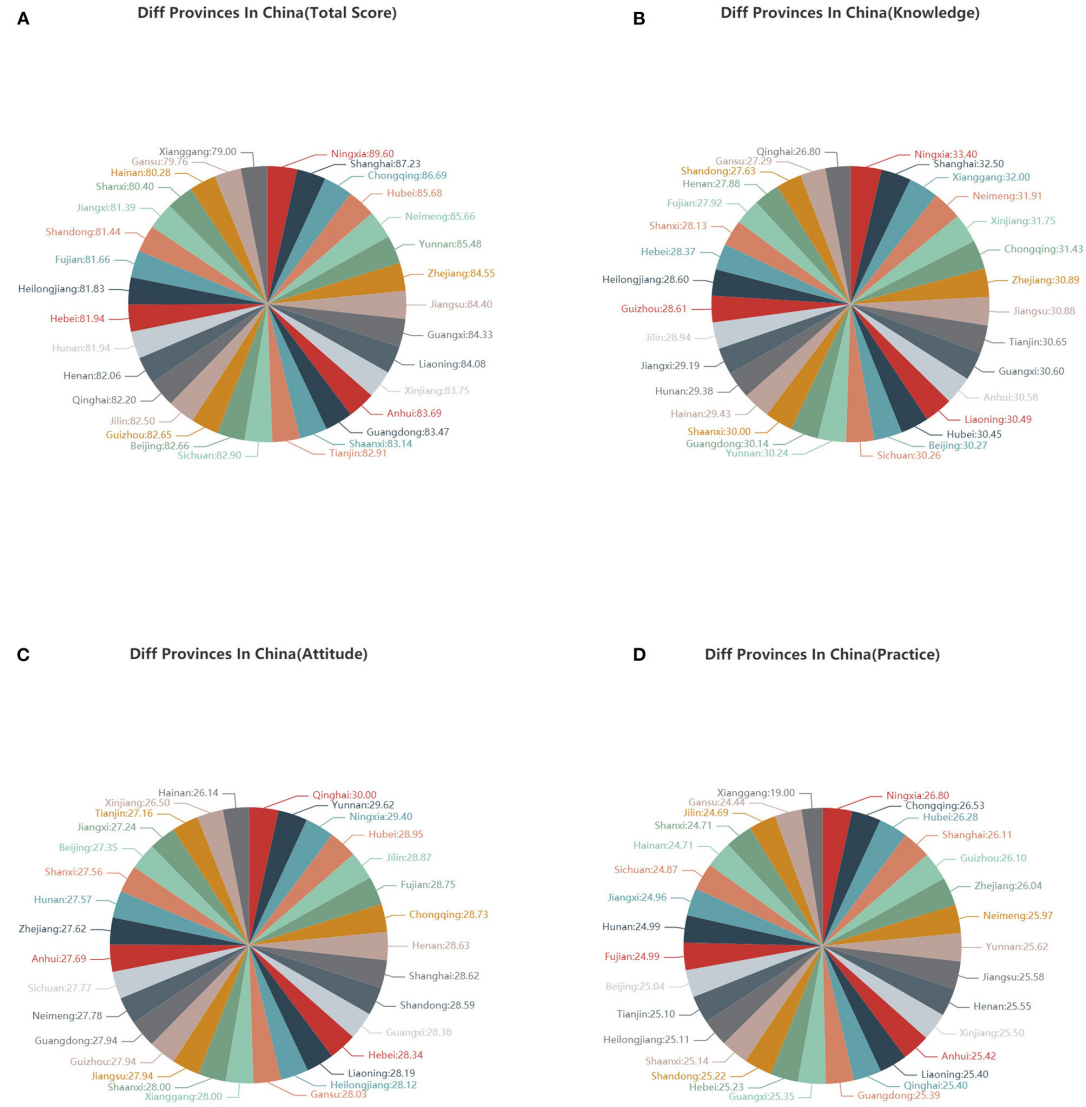
**(A)** The distributions of average KAP scores in different provinces in China by pie chart; **(B)** The distributions of average knowledge scores in different provinces in China by pie chart; **(C)** The distributions of average attitude scores in different provinces in China by pie chart; **(D)** The distributions of average practice scores in different provinces in China by pie chart.

The distributions of scores of knowledge, attitude and practice, were manifested by 3D scatter (Figure 1). To illustrate the distributions of scores among different provinces in China, the average scores of participants are illustrated by pie chart in Figure 2. Totally, the mean score of KAP was 83.3 ± 10.8, and fourteen provinces have higher scores than this, including Hubei province (Figure 2A). In an analysis according to each section of knowledge, attitude, and practice, the mean score was 28.4 ± 6.0, 28.2 ± 3.9, and 26.6 ± 4.1, respectively (Figures 2B–D).

Univariate and multivariate Logistic regression models were used to identify the risk of socio-characteristics of KAP (Table 1). The analysis highlighted that the older group had higher scores of KAP than those of the younger group (all *P* < 0.001). The participants of the ≥61 group had the highest Odds Ratio (OR) of 4.78 after adjustment for other variables (for instance, gender, education status, marital status, occupation, family member living together, family income, current status affected by COVID-19, and the appearance of clinical symptoms in the previous 14 days). Participants with higher educational levels (college/university and graduate or above) were subject to have higher scores on KAP than those with lower educational levels (OR_adj_ = 1.72, 95% CI 1.16–2.55, *P* = 0.01; OR_adj_ = 2.58, 95% CI 1.74–3.82, *P* < 0.001; OR_adj_ = 3.27, 95% CI 2.07–5.15, *P* < 0.001; for groups of senior, college/university, and graduate or above, respectively). In addition, those married participants, accounting for the largest proportions, showed higher scores on KAP (OR_adj_ = 1.18, 95% CI = 1.08–1.29, *P* < 0.001). Participants living with more than two family members were linked to higher scores in an increasing ORs when the family members increased (OR_adj_ = 1.28, 95% CI = 1.05–1.56, *P* = 0.01; OR_adj_ = 1.33, 95% CI = 1.10–1.60, *P* < 0.01; OR_adj_ = 1.27, 95% CI = 1.03–1.55, *P* = 0.02; for groups of 2, 3–4, and ≥5, respectively). Comparing with participants in low-income families, the 130,000–170,000 rmb group had the highest scores on KAP (OR_adj_ = 1.72, 95% CI 1.50–1.96, *P* < 0.001). The participants with the appearance of clinical symptoms in the previous 14 days, such as fever, cough, runny nose, accounting for 87.8% of participants, responded with an association with lower scores of KAPs (OR_adj_ = 0.67, 95% CI = 0.58–0.77, *P* < 0.001).

### Subgroup Analysis

For the stratified analyses (Tables 2–4), the associations between subgroups of socio-characteristics and scores on each section of knowledge, attitude, and practice were identified by univariate and multivariate Logistic regression models.

**Table 2:** Univariate and multivariate logistic regression analysis assessing scores of COVID-19 knowledge stratified by socio-demographic characteristics of residents, China.

	**SCORES**	**Univariate logistic regression**		**Multivariate logistic regression**	
	**(Mean ± SD)**	**OR (95% CI)**	***P*-value**	**OR (95% CI)**	***P*-value**
**Gender**					
Male	28.5 ± 6.5	1.00		1.00	
Female	28.3 ± 5.6	0.74 (0.68–0.80)	<0.0001	0.87 (0.80–0.95)	0.0202
**Age, years**					
≤ 20	26.8 ± 6.0	1.00		1.00	
21–40	28.7 ± 5.9	1.87 (1.64–2.14)	<0.0001	1.52 (1.30–1.77)	<0.0001
41–60	27.4 ± 6.6	1.29 (1.07–1.56)	0.008	1.42 (1.15–1.76)	0.001
≥61	29.3 ± 4.2	1.97(1.27–3.04)	0.002	3.29 (2.05–5.27)	<0.0001
**Education status**					
No formal education/Primary	21.7 ± 8.6	1.00		1.00	
Junior	24.7 ± 6.9	1.37(0.88–2.13)	0.158	1.39 (0.88–2.21)	0.162
Senior	27.1 ± 6.0	2.49 (1.64–3.79)	<0.0001	2.28 (1.46–3.55)	<0.0001
College/University	29.7 ± 5.2	5.50 (3.63–8.33)	<0.0001	4.19 (2.69–6.53)	<0.0001
Graduate or above	29.8 ± 6.4	8.14 (5.09–13.03)	<0.0001	5.73 (3.48–9.44)	<0.0001
**Marital status**					
Single	28.5 ± 6.0	1.00		1.00	
Married	28.6 ± 5.8	1.02 (0.94–1.10)	0.677	0.96 (0.87–1.05)	0.363
Divorced	24.2 ± 7.7	0.42 (0.31–0.58)	<0.0001	0.56 (0.40–0.79)	0.001
Widowed	20.2 ± 9.3	0.36 (0.17–0.77)	0.009	0.42 (0.18–0.98)	0.046
**Occupations**					
Managers of government/enterprise	28.8 ± 6.3	1.00		1.00	
Professionals	29.9 ± 6.0	1.47 (1.25–1.73)	<0.0001	1.49 (1.26–1.76)	<0.0001
Clerks	28.6 ± 6.7	1.00 (0.84–1.19)	0.960	1.00 (0.83–1.20)	0.972
Employees of commercial/service industry	28.3 ± 5.2	0.67 (0.58–0.77)	<0.0001	0.85 (0.74–0.99)	0.031
Workers in agriculture/forestry/animal husbandry/fishing/water conservancy	26.4 ± 8.3	0.75 (0.61–0.94)	0.012	1.16 (0.92–1.47)	0.216
Operators of production/transportation equipment	29.5 ± 6.7	1.47 (1.19–1.83)	<0.0001	1.66 (1.33–2.07)	<0.0001
Polices/Militaries/Guards	23.4 ± 7.9	0.30 (0.15–0.58)	<0.0001	0.41 (0.20–0.82)	0.009
Others	27.7 ± 5.2	0.56 (0.47–0.66)	<0.0001	0.79 (0.66–0.94)	0.009
Unemployed	27.4 ± 5.9	0.59 (0.50–0.69)	<0.0001	1.05 (0.87–1.27)	0.601
**Family members living together (No.)**					
Single	27.6 ± 6.2	1.00		1.00	
1	26.2 ± 6.8	0.73 (0.56–0.95)	0.019	0.77 (0.58–1.03)	0.079
2	28.6 ± 6.5	1.50 (1.25–1.80)	<0.0001	1.33 (1.09–1.63)	0.005
3–4	28.6 ± 5.9	1.35 (1.14–1.61)	0.001	1.21 (1.00–1.46)	0.047
≥5	28.3 ± 5.5	1.13 (0.94–1.36)	0.195	1.10 (0.89–1.35)	0.368
**Family incomes (rmb per year)**					
<50,000	26.4 ± 6.1	1.00		1.00	
50,000–120,000	29.0 ± 5.5	2.36 (2.14–2.60)	<0.0001	1.85 (1.66–2.05)	<0.0001
130,000–170,000	29.8 ± 5.7	3.21 (2.84–3.64)	<0.0001	2.34 (2.04–2.68)	<0.0001
180,000–250,000	29.6 ± 6.4	3.37 (2.89–3.93)	<0.0001	2.37(2.01–2.79)	<0.0001
>250,000	28.6 ± 6.6	2.41 (2.00–2.90)	<0.0001	1.71(1.40–2.09)	<0.0001
**Current status affected by COVID-19**					
Diagnosed, and cured	18.9 ± 9.6	1.00		1.00	
Diagnosed, and under treatment	16.3 ± 8.3	0.58 (0.17–1.98)	0.380	0.43 (0.12–1.52)	0.188
Suspected, and quarantined	20.0 ± 9.9	2.03 (0.80–5.14)	0.135	1.48 (0.56–3.88)	0.427
Home-based quarantine	23.4 ± 9.7	2.63 (1.10–6.22)	0.028	2.11 (0.86–5.17)	0.102
Confirmed healthy after quarantine	29.6 ± 6.2	8.71 (3.76–20.18)	<0.0001	7.23 (3.01–17.35)	<0.0001
None of above	28.7 ± 5.6	4.49 (1.99–10.17)	<0.0001	4.06 (1.73–9.56)	0.001
Appearance of clinical symptoms in previous 14 days^*^					
No	28.7 ± 5.5	1.00		1.00	
Yes	26.1 ± 8.5	0.90 (0.79–1.01)	0.066	0.91 (0.79–1.05)	0.200

*OR, odds ratio; CI, confidence interval*.

* *Referred to symptoms of fever, cough, expectoration, diarrhea, weak, headache, runny nose, rhinobyon, sore throat*.

**Table 3:** Univariate and multivariate logistic regression assessing scores of attitudes toward COVID-19 by socio-demographic characteristics of residents.

	**Attitudes**	**Univariate logistic regression**		**Multivariate logistic regression**	
	**(Mean ± SD)**	**OR (95% CI)**	***P*-value**	**OR (95% CI)**	***P*-value**
**Gender**					
Male	27.9 ± 4.2	1.00		1.00	
Female	28.5 ± 3.7	1.20 (1.11–1.30)	<0.0001	1.14 (1.05–1.24)	0.011
**Age, years**					
≤ 20	26.6 ± 4.1	1.00		1.00	
21–40	28.4 ± 3.8	2.29 (1.98–2.66)	<0.0001	1.92 (1.63–2.25)	<0.0001
41–60	28.8 ± 3.8	2.70 (2.22–3.29)	<0.0001	2.25 (1.82–2.79)	<0.0001
≥61	28.7 ± 3.5	2.15 (1.37–3.36)	0.001	2.09 (1.31–3.34)	0.002
**Education status**					
No formal education/Primary	26.0 ± 6.6	1.00		1.00	
Junior	28.2 ± 4.3	1.31 (0.90–1.89)	0.157	1.00 (0.68–1.24)	0.997
Senior	28.3 ± 3.9	1.31 (0.92–1.86)	0.138	1.06 (0.73–1.55)	0.746
College/University	28.3 ± 3.7	1.27 (0.90–1.80)	0.178	1.00 (0.69–1.45)	0.994
Graduate or above	28.1 ± 4.0	1.37 (0.91–2.06)	0.129	1.13 (0.73–1.74)	0.587
**Marital status**					
Single	27.8 ± 4.0	1.00		1.00	
Married	28.6 ± 3.8	1.40 (1.29–1.52)	<0.0001	1.16 (1.06–1.27)	0.002
Divorced	27.7 ± 4.6	1.19 (0.88–1.60)	0.257	1.15 (0.85–1.57)	0.369
Widowed	24.5 ± 6.2	0.46 (0.20–1.05)	0.066	0.48 (0.20–1.14)	0.096
**Occupations**					
Managers of government/enterprise	28.0 ± 4.3	1.00		1.00	
Professionals	28.3 ± 4.0	1.13 (0.97–1.33)	0.128	1.12 (0.95–1.32)	0.188
Clerks	28.0 ± 4.0	0.99 (0.83–1.18)	0.903	0.96 (0.80–1.15)	0.629
Employees of commercial/service industry	28.7 ± 3.6	1.24 (1.08–1.42)	0.003	1.18 (1.02–1.37)	0.022
Workers in agriculture/forestry/animal husbandry/fishing/water conservancy	27.3 ± 4.6	0.85 (0.67–1.07)	0.156	0.87 (0.68–1.10)	0.230
Operators of production/transportation equipment	28.6 ± 3.7	1.19 (0.97–1.47)	0.005	1.14 (0.92–1.41)	0.221
Polices/Militaries/Guards	25.7 ± 4.1	0.33 (0.15–0.71)	0.01	0.46 (0.21–1.02)	0.056
Others	28.4 ± 3.7	1.10 (0.93–1.31)	0.271	1.04 (0.87–1.24)	0.705
Unemployed	27.4 ± 3.9	0.70 (0.59–0.83)	<0.0001	0.75 (0.62–0.91)	0.003
**Family members living together (No.)**					
Single	27.4 ± 4.6	1.00		1.00	
1	27.7 ± 4.5	1.13 (0.86–1.49)	0.369	1.06 (0.80–1.40)	0.702
2	28.0 ± 3.9	1.32 (1.08–1.59)	0.005	1.23 (1.01–1.50)	0.043
3–4	28.3 ± 3.8	1.47 (1.23–1.76)	<0.0001	1.34 (1.11–1.62)	0.002
≥5	28.6 ± 3.7	1.60 (1.32–1.95)	<0.0001	1.40(1.14–1.72)	0.001
**Family incomes (rmb per year)**					
<50,000	28.1 ± 4.1	1.00		1.00	
50,000–120,000	28.3 ± 3.7	1.01 (0.91–1.11)	0.884	0.94 (0.85–1.04)	0.226
130,000–170,000	28.2 ± 4.0	1.00 (0.88–1.23)	0.975	0.97 (0.85–1.10)	0.606
180,000–250,000	28.5 ± 3.7	1.17 (1.00–1.35)	0.045	1.12 (0.95–1.31)	0.173
>250,000	28.5 ± 4.1	1.26 (1.04–1.51)	0.016	1.21 (1.00–1.47)	0.052
**Your current status affected by COVID-19**					
Diagnosed, and cured	21.3 ± 7.1	1.00		1.00	
Diagnosed, and under treatment	20.6 ± 4.4	0.21 (0.04–1.10)	0.065	0.21 (0.04–1.09)	0.063
Suspected, and quarantined	23.8 ± 4.5	0.63(0.23–1.77)	0.383	0.59 (0.21–1.67)	0.318
Home-based quarantine	25.7 ± 4.8	1.40 (0.59–3.36)	0.448	1.19 (0.49–2.87)	0.702
Confirmed healthy after quarantine	28.2 ± 3.9	3.24 (1.40–7.50)	0.006	2.70 (1.15–6.29)	0.022
None of above	28.4 ± 3.7	3.24 (1.43–7.32)	0.005	2.34 (1.02–5.36)	0.045
Appearance of clinical symptoms in previous 14 days^*^					
No	28.5 ± 3.7	1.00		1.00	
Yes	26.6 ± 4.9	0.65 (0.57–0.74)	<0.0001	0.80 (0.69–0.92)	0.002

* *Referred to symptoms of fever, cough, expectoration, diarrhea, weak, headache, runny nose, rhinobyon, sore throat*.

**Table 4:** Univariate and multivariate logistic regression assessing scores of practices regarding COVID-19 by socio-demographic characteristics of residents.

	**Practices**	**Univariate logistic regression**		**Multivariate logistic regression**	
	**(Mean ± SD)**	**OR (95% CI)**	***P*-value**	**OR (95% CI)**	***P*-value**
**Gender**					
Male	26.3 ± 4.5	1.00		1.00	
Female	26.9 ± 3.7	1.07 (0.98–1.16)	0.115	1.05 (0.97–1.14)	0.252
**Age, years**					
≤ 20	25.3 ± 4.5	1.00		1.00	
21–40	26.8 ± 4.0	1.91 (1.66–2.21)	<0.0001	1.54 (1.31–1.80)	<0.0001
41–60	26.8 ± 4.1	1.84 (1.51–2.24)	<0.0001	1.54 (1.24–1.90)	<0.0001
≥61	27.1 ± 3.2	1.87 (1.19–2.92)	0.006	1.93 (1.21–3.10)	0.006
**Education status**					
No formal education/Primary	23.2 ± 6.2	1.00		1.00	
Junior	25.9 ± 4.8	1.83 (1.22–2.75)	0.004	1.39 (0.91–2.13)	0.138
Senior	26.4 ± 4.3	1.97 (1.33–2.91)	0.001	1.50 (0.9–2.26)	0.060
College/University	26.9 ± 3.8	2.14 (1.45–3.15)	<0.0001	1.50 (0.99–2.26)	0.058
Graduate or above	26.5 ± 4.5	2.29 (1.47–3.56)	<0.0001	1.67 (1.05–2.66)	0.035
**Marital status**					
Single	26.3 ± 4.1	1.00		1.00	
Married	27.0 ± 3.9	1.32 (1.22–1.44)	<0.0001	1.17 (1.06–1.28)	0.001
Divorced	24.3 ± 6.2	0.78 (0.57–1.08)	0.130	0.86 (0.62–1.19)	0.358
Widowed	18.8 ± 7.5	0.11 (0.03–0.47)	0.003	0.15 (0.03–0.62)	0.009
**Occupations**					
Managers of government/enterprise	26.5 ± 4.2	1.00		1.00	
Professionals	26.9 ± 4.1	1.23 (1.05–1.44)	0.011	1.19 (1.01–1.40)	0.035
Clerks	26.5 ± 4.4	1.08 (0.91–1.29)	0.387	1.03 (0.86–1.23)	0.769
Employees of commercial/service industry	27.0 ± 3.7	1.06 (0.92–1.22)	0.425	1.03 (0.89–1.19)	0.692
Workers in agriculture/forestry/animal husbandry/fishing/water conservancy	25.1 ± 5.7	0.87 (0.70–1.10)	0.248	0.96 (0.75–1.21)	0.756
Operators of production/transportation equipment	26.9 ± 4.1	1.18 (0.96–1.46)	0.111	1.13 (0.92–1.40)	0.257
Polices/Militaries/Guards	22.6 ± 5.9	0.28 (0.12–0.63)	0.002	0.38 (0.17–0.88)	0.023
Others	27.0 ± 3.6	0.95 (0.80–1.13)	0.548	0.91 (0.76–1.09)	0.335
Unemployed	25.9 ± 4.2	0.67 (0.57–0.80)	<0.0001	0.75 (0.62–0.91)	0.003
**Family members living together (No.)**					
Single	25.8 ± 4.5	1.00		1.00	
1	25.9 ± 4.9	0.96 (0.73–1.27)	0.776	0.93 (0.70–1.23)	0.609
2	26.4 ± 4.4	1.18 (0.98–1.43)	0.089	1.06 (0.87–1.29)	0.589
3–4	26.8 ± 4.0	1.38 (1.15–1.65)	<0.0001	1.20 (1.00–1.45)	0.056
≥5	27.0 ± 3.8	1.37 (1.13–1.66)	0.002	1.18 (0.96–1.45)	0.118
**Family incomes (rmb per year)**					
<50,000	26.2 ± 4.2	1.00		1.00	
50,000–120,000	25.0 ± 3.4	1.24 (1.13–1.37)	<0.0001	1.13 (1.02–1.26)	0.023
130,000–170,000	26.9 ± 3.7	1.45 (1.28–1.64)	<0.0001	1.34 (1.17–1.53)	<0.0001
180,000–250,000	26.8 ± 4.2	1.33 (1.14–1.54)	<0.0001	1.20 (1.02–1.41)	0.026
>250,000	26.5 ± 4.7	1.13 (0.94–1.37)	0.205	1.04 (0.85–1.27)	0.696
**Current status affected by COVID-19**					
Diagnosed, and cured	18.9 ± 5.4	1.00		1.00	
Diagnosed, and under treatment	17.7 ± 4.6	0.79 (0.15–4.19)	0.780	0.90 (0.12–4.22)	0.88
Suspected, and quarantined	20.4 ± 6.8	2.56 (0.70–9.35)	0.154	2.58 (0.7–9.53)	0.154
Home-based quarantine	22.7 ± 6.5	3.30 (0.97–11.18)	0.055	2.76 (0.80–9.44)	0.107
Confirmed healthy after quarantine	26.5 ± 4.7	8.65 (2.62–28.51)	<0.0001	6.70 (2.01–22.32)	0.002
None of above	26.9 ± 3.8	8.09 (2.49–26.25)	0.001	4.57 (1.39–15.02)	0.012
Appearance of clinical symptoms in previous 14 days^*^					
No	27.0 ± 3.7	1.00		1.00	
Yes	24.0 ± 5.7	0.45 (0.39–0.51)	<0.0001	0.48 (0.41–0.57)	<0.0001

* *Referred to symptoms of fever, cough, expectoration, diarrhea, weak, headache, runny nose, rhinobyon, sore throat*.

By comparison, the participants in the ≥61 year age group were linked to higher scores on knowledge, attitude and practice (all *P*_adj_ < 0.01). Participants with college/university and graduate/above education had significantly greater awareness and practice (all *P*_adj_ < 0.01). The subgroups of occupational types, professionals and operators of production/transportation equipment, both of which tended to achieve higher scores of knowledge (both *P*_adj_ < 0.01).

From the study, it was revealed that the married and participants living with more than five family members were prone to achieve higher scores of knowledge, attitude and practice than other groups (all *P*_adj_ < 0.01). In the subgroup analysis of family incomes, participants of 130,000–170,000 groups achieved higher groups of knowledge and practice (both *P*_adj_ < 0.01).

During the epidemic of COVID-19, those participants confirmed healthy after quarantine and those without quarantine or diagnosis were linked to higher score on knowledge, attitude and practice (all *P*_adj_ < 0.01). Those participants without any clinical symptoms, such as fever, cough, runny nose, and sputum, were prone to achieve higher scores of attitude and practices (all *P*_adj_ < 0.01).

### CART Model Construction

**Figure 3:**
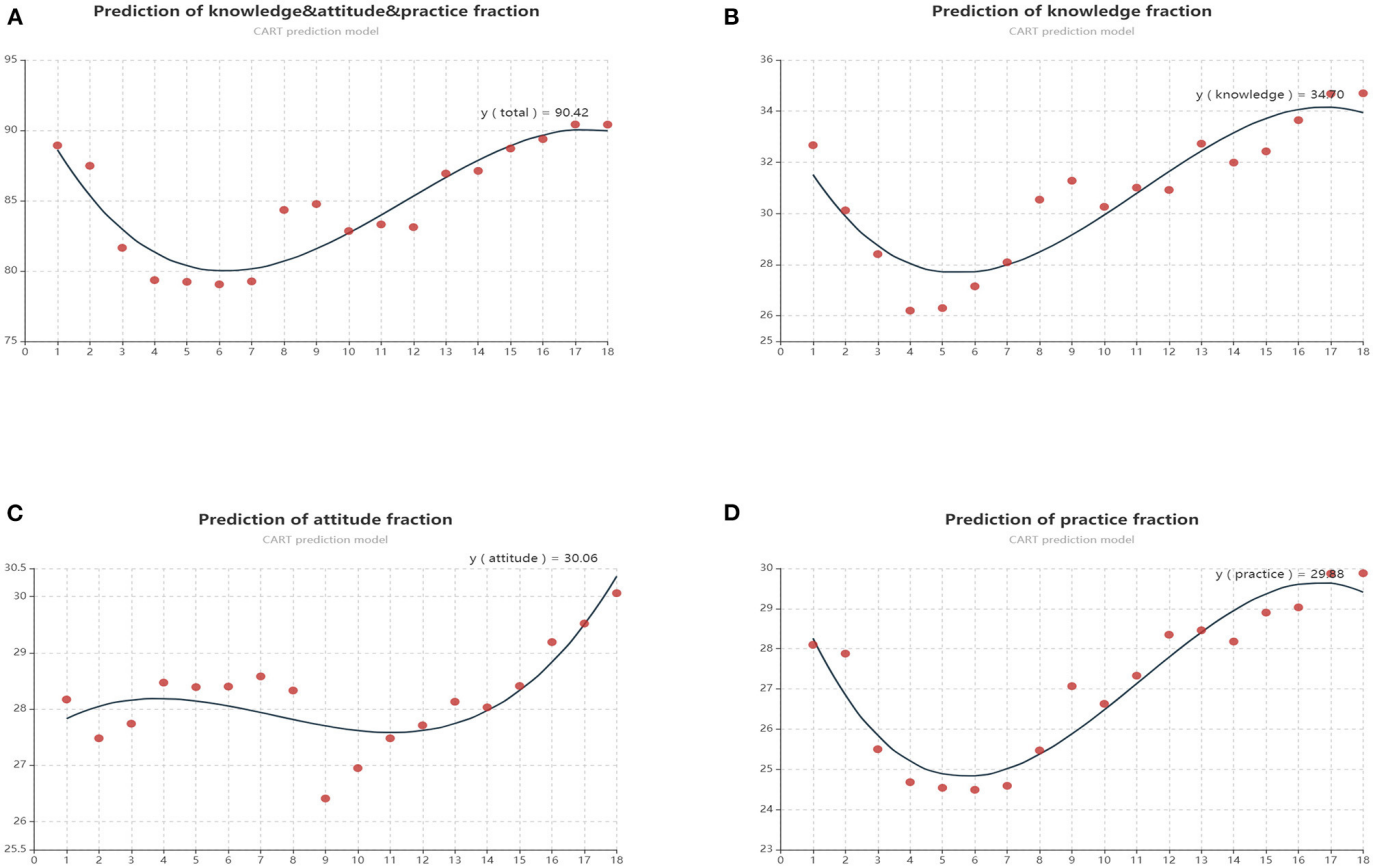
**(A)** Predictive scores of KAP calculated by CART model; **(B)** Predictive scores of knowledge scores calculated by CART model; **(C)** Predictive scores of attitude scores calculated by CART model; **(D)** Predictive scores of practice scores calculated by CART model.

Additionally, a CART model was used to build predication relationships between answer time of completing questionnaires and scores of KAP (Figure 3A). The CART procedure was done in the model by building a set of participants using the answer time of the questionnaire as a potential predictor. CART selected a peak cutoff score of 90.4 for no further evaluation. Moreover, analysis revealed that the predictive scores of knowledge, attitude and practice section was 34.7, 30.1, and 29.9, respectively (Figures 3B–D).

## Discussion

During the epidemic of COVID-19, we used a random sampling method to assess residents' knowledge, attitudes, and practice behaviors toward COVID-19 in 32 provinces of China. Overall, a better response toward COVID-19 accrued from the participants who were married, those with middle family income, and those who lived with more than two family members. The majority of these participants were able to recognize symptoms and the transmission risk of COVID-19.

During the Chinese Spring Festival, travel bans, lockdowns and movement restrictions were implemented across the whole nation, which disproportionately affected the residents who were without sufficient social and family support, including those who were homeless, incarcerated, migrants, or refugees (9, 10). Those residents might not have regular access to basic hygiene knowledge or supplies, which made them susceptible to virus transmission. During the epidemic, the vast majority of residents chose to stay at home with their family members, which created more opportunities and time to care and support for each other. Survey results revealed that the married participants and those living with more than two family members received better social and family support, because it appeared not only to increase positive mental health-related lifestyle changes (11, 12), but also be conducive to health education.

Furthermore, the results of this study indicated that both educational levels and family incomes were linked to the cognition of COVID-19. Participants with higher than college/university educational levels, accounting for the largest proportions of participants, and participants with family income of 130,000**–**170,000 rmb per year, both displayed a better response to knowledge and practice toward COVID-19. These residents make up the core workforce in China, and also were the very populations most affected by enforcement of movement bans and quarantines. In addition, there is no age-group protection from COVID-19, however, the most severe cases were more than 70 years old, with a mortality rates of more than 20% among octogenarians (13, 14). Due to their inaccessibility to mobile software, the participants aged more than 60 years old only accounted for 0.9% of the study.

During the outbreak of COVID-19, in addition to the Wuhan lockdown area, several compulsive measures were implemented to respond to the national emergency. For example, prohibition of public gatherings and entertainment, shutdown of factories and schools, quarantine and isolation, restriction of access to residential areas, all of these changed lifestyles and patterns drastically in every aspect of daily life (15). Travel bans and isolation were the first response to new infectious disease, enforcing thousands of residents who had been exposed to COVID-19 to isolation and self-quarantine. But coercive measures could be counterproductive and erode public trust and cooperation (16). Therefore, it is of great importance to identify the awareness and attitudes of residents who experienced the period of quarantine or isolation. In the present study, participants who were confirmed as healthy after 14 days of quarantine, and those who were not exposed to and not infected by COVID-19, displayed a better response to the survey. The vast majority of participants showed their satisfaction and faith in the measures taken by the authorities during the epidemic. To further explore their attitude toward authorities, we found that those holding a positive attitude also responded better on knowledge regarding COVID-19; while those holding a less positive attitude also practiced worse behaviors or protections, which seemed to account for their faith and support in health authorities in return.

Meanwhile, faced with an overwhelming national pandemic, residents' behaviors toward COVID-19 were of great importance. Scientific behaviors for protection were, therefore, of critical importance, requiring the rapid and appropriate behavioral changes to reduce transmission of disease. In the study, the vast majority of residents had gained insights into the necessity of wearing a mask during the epidemic. It was also demonstrated that surgical face masks could reduce the emission of influenza virus into the environment in terms of respiratory droplets (17), indicating its potential effect for control of COVID-19. In a previous study conducted by Geldsetzer, 37.8% of US participants and 29.7% of UK participants declared that wearing a mask was highly effective to protect themselves from COVID-19-infected (18). However, it was revealed that 98.0% of residents in the Wuhan area would wear a mask when leaving the home during the outbreak of COVID-19 (19). Under the guidance by WHO (20, 21), there are still several suggestions on wearing a mask by public health. Firstly, it is essential to wear a mask in the hospital whether for visiting or for treatment. Secondly, the customers and the staff of public traffic vehicles, such as airplanes, buses and taxis should also wear a mask in daily life. Last but not least, the crowed places without appropriate ventilation, including banks, barbershops, supermarkets, restaurants, are the primary target places to wear a mask when going to these places. In China, however, messaging has advised residents that not wearing a mask is acceptable when staying in a well-ventilated home and in the open air without crowd. This assumption is still controversial around the world and changes of behaviors on its acceptance are worthy of expectations.

This study has at least two main limitations. First, the selection of residents within the nation was randomly selected by network, inducing potential selective bias. Although the survey covered areas with varying levels of COVID-19 incidence and in several provinces, it was not representative of all the nation. Second, the questionnaire used was not a standardized form, composed of single-choice and multiple-choice. To avoid this limitation, the scores were ruled and calculated by an expert panel.

## Conclusions and Policy Implications

During the epidemic of COVID-19, we found that participants who were older, married, with middle family income, and who lived with more than two family members, responded well to the survey, and the vast majority of respondents had faith in the measures adopted by the government and supported the measures used by the authorities, which might result from their better awareness and practices. Further research is still needed among a larger sample, such as health professionals, nurses, and confirmed patients. In addition, based on the previous experiences and lessons deriving from China, the following recommendations for daily protection could be proposed in order to prevent and contain the pandemic of COVID-19 in other countries. Specifically:

- Centralized quarantine and household quarantine for suspected cases have been acknowledged as primary and effective measures to curb the epidemic.
- The control measures enacted by authorities are crucial, such as forbidding public gatherings, shutdown of factories and schools, maintaining social distance, and controlling access to communities.
- As a daily effective measure during the epidemic, it is recommended to properly wear a face mask, and that it be properly disposed of after use.

Large scale research is necessary involving healthcare providers, nurses, and affected patients to confirm the validity of our survey and the protective and preventive suggestions we propose.

## Data Availability Statement

The original contributions presented in the study are included in the article/Supplementary Material, further inquiries can be directed to the corresponding author/s.

## Ethics Statement

The study was conducted according to the principles of Helsinki declaration. The bioethical committees at Fujian Medical University 2nd Affiliated Hospital, China, gave written approval for the study (2020-206).

## Author Contributions

YMZ conceived the study. YX, GFL, and CS made the statistics and figures. HFZ and SF designed the questionnaire. XYC, YFC, YXZ, and GAM consulted on the knowledge of COVID-19. XHZ, XJY, and LZ consulted on the figures. All authors interpreted the results, and contributed to writing the article. All authors approved the final version for submission.

## Conflict of Interest

The authors declare that the research was conducted in the absence of any commercial or financial relationships that could be construed as a potential conflict of interest.
