# Supplementary material for: Public Knowledge, Attitudes, and Practices Behaviors Towards Coronavirus Disease 2019 (COVID-19) During a National Epidemic—China
Source: Front Public Health. 2021 Mar 19;9:638430. doi: 10.3389/fpubh.2021.638430 (PMC8017307; doi:10.3389/fpubh.2021.638430)
Supplement: Supplementary file 1 [file Table_1.DOCX]

Supplementary Material

| **Table S1. Knowledge of COVID-19 among participants, China** | | | |
| --- | --- | --- | --- |
| Items | Response format | No. | % (95%CI) |
| **1. Perceptions about COVID-19** | multiple-choice |  |  |
| The source of infection mainly derives in confirmed patients |  | 8853 | 86.8 (86.2-87.5) |
| Asymptomatic carriers could be the source of infection |  | 8731 | 85.6 (85.0-86.3） |
| The disease spreads through the respiratory tract and close contact |  | 9722 | 95.4 (94.9-95.8) |
| The disease could spread by aerosol |  | 7341 | 72.0 (71.2-72.9) |
| **2. Perceptions of incubation period of COVID-19 based on the present investigations** | single-choice |  |  |
| 1-2 days |  | 86 | 0.8 (0.7-1.0） |
| 8-14 days |  | 2164 | 21.2 (20.4-22.0) |
| 1-14 days |  | 5362 | 52.6 (51.7-53.6) |
| More than 14 days |  | 2583 | 25.3 (24.5-26.2) |
| **3. Perceptions of the definition on “close contacts”** | multiple-choice |  |  |
| Family members living in the same room |  | 9519 | 93.4 (92.9-93.8) |
| Colleagues in the same office |  | 9326 | 91.5 (90.9-92.0) |
| Roommates in the same dorm |  | 9205 | 90.3 (89.7-90.8) |
| Persons using the same elevator together |  | 9056 | 88.8 (88.2-89.4) |
| Persons with dinner together |  | 9519 | 93.4 (92.9-93.9) |
| **4. Perceptions about the typical clinical symptoms of COVID-19** | multiple-choice |  |  |
| Fever and cough |  | 9830 | 96.4 (96.0-96.8) |
| Diarrhea |  | 3830 | 37.6 (36.6-38.6) |
| Chest stuffiness and dyspnea |  | 5152 | 50.5 (49.5-51.5) |
| Coma |  | 2855 | 28.0 (27.1-28.9) |
| **5. Perceptions after closely contacting with confirmed COVID-19 patients** | single-choice |  |  |
| Centralized quarantine under medical observation |  | 730 | 7.2 (6.7-7.7) |
| Household quarantine |  | 610 | 6.0 (5.6-6.4) |
| Inform of managing organization after the development of respiratory symptom |  | 491 | 4.8 (4.4-5.2) |
| All of above |  | 8364 | 82.0 (81.3-82.7) |
| **6. Attentions need to be paid during the course of medical treatment if I had a fever** | single-choice |  |  |
| Choose hospital randomly |  | 230 | 2.3 (2.0-2.6) |
| Do not need to wear a mask before diagnosis |  | 150 | 1.5 (1.2-1.7) |
| Go to designated hospital by bus |  | 259 | 2.5 (2.2-2.8) |
| Inform of doctors about the history of traveling and contacting with Wuhan area |  | 9556 | 93.7 (93.2-94.2) |
| **7. Preventions has been used to curb the spread of COVID-19 in public places** | single-choice |  |  |
| Avoid to go crowed places, such as market, subway |  | 578 | 5.7 (5.2-6.1) |
| Wear a mask |  | 869 | 8.5 (8.0-9.1) |
| Go to hospital after having a fever appears |  | 192 | 1.9 (1.6-2.1) |
| All of above |  | 8556 | 83.9 (83.2-84.7) |
| **8. Perceptions of advised personal measures used for preventing COVID-19** | Multiple -choice |  |  |
| Visit doctors frequently |  | 3521 | 34.5 (33.6-35.5) |
| Wash hands with soap and water frequently |  | 9810 | 96.2 (95.8-96.6) |
| Open the door for ventilation frequently |  | 9500 | 93.2 (92.7-93.7) |
| Reduce the time of staying in crowed places |  | 9640 | 94.6 (94.1-95.0) |
| **9. The preventive measures taken by the government** | multiple-choice |  |  |
| Control and forbid the crowed in public places, such as market, cinema |  | 9524 | 93.4 (92.9-93.9) |
| Suspend of work and school |  | 9546 | 93.6 (93.1-94.1) |
| Access control of community residents |  | 9617 | 94.3 (93.9-94.7) |
| Stop the killings of wildlife and poultry |  | 8552 | 83.9 (83.1-84.6) |
|  |  |  |  |

| **Table S2. Attitudes of COVID-19 among participants, China** |  |  | |  |
| --- | --- | --- | --- | --- |
| Items | Response format | | No. | % (95%CI) |
| **1. COVID-19 had a serious influence on life** | single-choice | |  |  |
| Strongly disagree |  | | 142 | 1.4 (1.2-1.6) |
| Disagree |  | | 329 | 3.2 (2.9-3.6) |
| Neutral |  | | 1612 | 15.8 (15.1-16.5) |
| Agree |  | | 4861 | 47.7 (46.8-48.6) |
| Strongly agree |  | | 3251 | 31.9 (31.0-32.8) |
| **2. I am worried about infecting with COVID-19** | single-choice | |  |  |
| Never worried |  | | 1022 | 10.0 (9.5-10.6) |
| No worried |  | | 1996 | 19.6 (18.8-20.3) |
| A little |  | | 5222 | 51.2 (50.3-52.2) |
| Very worried |  | | 1955 | 19.2 (18.4-20.0) |
| **3. I would be more nervous than ever if I caught a fever or cough** | single-choice | |  |  |
| Strongly disagree |  | | 998 | 9.8 (9.2-10.4) |
| Disagree |  | | 340 | 3.3 (3.0-3.7) |
| Neutral |  | | 1390 | 13.6 (12.9-14.3) |
| Agree |  | | 5358 | 52.6 (51.6-53.5) |
| Strongly agree |  | | 2109 | 20.7 (19.9-21.5） |
| **4. I focus on the information on COVID-19** | single-choice | |  |  |
| No at all |  | | 147 | 1.4 (1.2-1.7) |
| Less concerned, and pay no attention to the related articles |  | | 609 | 6.0 (5.5-6.4) |
| More concerned, and read several related articles |  | | 4390 | 43.1 (42.1-43.9) |
| Very concerned, and be familiar with the growth dynamic in China |  | | 5049 | 49.5 (48.6-50.7) |
| **5. The disease will become widespread around the nation** | single-choice | |  |  |
| Yes, globalization would accelerate its transmission dramatically |  | | 1352 | 13.3 (12.6-13.9) |
| May be like the situation of Severe Acute Respiratory Syndromes (SARS) |  | | 1049 | 10.3 (9.7-10.8) |
| Unlikely, China has taken several measures to resolve the problem |  | | 3383 | 33.2 (32.3-34.1) |
| Impossible, I believe that the preventive measures would curb the outbreak |  | | 4411 | 43.3 (42.3-44.3) |
| **7. I am satisfied with the control measures administrated by the government** | single-choice | |  |  |
| Strongly disagree |  | | 319 | 3.1 (2.8-3.5) |
| Disagree |  | | 120 | 1.2 (1.0-1.4) |
| Neutral |  | | 989 | 9.7 (9.1-10.3) |
| Agree |  | | 4671 | 45.8 (44.8-46.8) |
| Strongly agree |  | | 4096 | 40.2 (39.2-41.1) |
| **8. The faith of the control measures used by the government** | multiple-choice | |  |  |
| Have strong confidence, because China has used sufficient quarantine measures |  | | 6608 | 64.8 (63.9-65.7) |
| Have confidence, accompanying with some scares due to the severity of epidemic |  | | 2581 | 25.3 (24.5-26.1) |
| Be more warried, being still under the influence of SARS |  | | 395 | 3.9 (3.5-4.2) |
| Be very warried, it would not curb the outbreak using the existing measures |  | | 611 | 6.0 (5.6-6.4) |
| **9. I would support the measures taken by the local government to curb the outbreak** | single-choice | |  |  |
| Strongly supported |  | | 7774 | 76.3 (75.4-77.1) |
| More supported |  | | 2224 | 21.8 (21.0-22.6) |
| Be skeptical |  | | 85 | 0.8 (0.7-1.0) |
| Not supported |  | | 112 | 1.1 (0.9-1.3) |

| **Table S3. Practice of COVID-19 among participants, China** |  |  |  |
| --- | --- | --- | --- |
| Items | Response format | No. | % (95%CI) |
| **1.Measures used to spend this year's Lunar New Year holidays** | single-choice |  |  |
| Same as previous years |  | 323 | 3.2 (2.9-3.5) |
| Stayed at home |  | 9310 | 91.3 (90.8-91.8） |
| Played outside，especially in crowded places |  | 363 | 3.6 (3.2-3.9) |
| Played outside，but avoided from crowed places |  | 199 | 2.0 (1.7-2.2) |
| **2. Intentions after being invited by friends or relatives to go outings** | single-choice |  |  |
| Would not go out |  | 8714 | 85.5 (84.7-86.2) |
| It depends |  | 1090 | 10.7 (10.1-11.3) |
| Uncertain |  | 272 | 2.7 (2.4-3.0) |
| Would go out |  | 119 | 1.2 (1..0-1.4） |
| **3. Intentions if family member or friends had a fever, cough** | single-choice |  |  |
| Not to matter |  | 102 | 1.0 (0.8-1.2) |
| Conceal the information |  | 150 | 1.5 (1.3-1.7) |
| Take protective measures for fear of infection |  | 1583 | 15.5 (14.9-16.3) |
| Persuade him/her to see a doctor |  | 8360 | 82.0 (81.3-82.7) |
| **4. Disposal measures of a used mask** | single-choice |  |  |
| Put it into the designated dustbin in the community |  | 6433 | 63.1 (62.2-64.0) |
| Throw it at will |  | 201 | 2.0 (1.7-2.3) |
| Dispose it after surface sterilization |  | 2940 | 28.8 (28.0-29.7) |
| Reuse it after surface sterilization |  | 621 | 6.1 (5.6-6.5) |
| **5. Measures used to contact with friends** | multiple-choice |  |  |
| Contact through social software, such as WeChat, QQ |  | 9478 | 93.0 (92.5-93.5) |
| Contact through telephone |  | 8268 | 81.1 (80.3-81.8) |
| Meet face to face |  | 521 | 5.1 (4.7-5.5) |
| Be out of touch |  | 1094 | 10.7 (10.1-11.3) |
| **6. Practices used when you must meet your friends** | multiple-choice |  |  |
| Wear a mask |  | 9654 | 94.7 (94.3-95.1) |
| Shake hands |  | 1117 | 11.0 (10.3-11.6) |
| Keep 1-meter distance from each other |  | 8958 | 87.9 (87.2-88.5) |
| No protective measures |  | 257 | 2.5 (2.2-2.8) |
| **7. Personal protective measures used during the epidemic** | multiple-choice |  |  |
| Open window for ventilation |  | 9426 | 92.4 (91.9-93.0) |
| No protective measures |  | 1027 | 10.1 (9.4-10.7) |
| Do household quarantine with less out of the door |  | 9489 | 93.1 (92.6-93.5) |
| Wear a face mask when going out |  | 9602 | 94.2 (93.7-94.6) |
| Take self-test temperature daily |  | 7631 | 74.8 (74.0-75.6） |
| **8. Measures used to buy vegetables** | multiple-choice |  |  |
| Eat out with no need for shopping |  | 699 | 6.9 (6.4-7.4) |
| Order a takeaway |  | 1517 | 14.9 (14.2-15.6） |
| Shop daily from a market or supermarket |  | 4793 | 47.0 (46.0-48.0) |
| Order online followed by being delivered home |  | 6054 | 59.4 (58.4-60.3) |
| Purchased through communities together |  | 3699 | 36.3 (35.4-37.3) |
| **9. Vehicles commonly used to travel** | multiple-choice |  |  |
| Public vehicles, such as bus, subway |  | 1170 | 11.5 (10.8-12.1) |
| Taxi or online car-hailing |  | 987 | 9.7 (9.1-10.2) |
| Shared bicycle |  | 1793 | 17.6 (16.9-18.3) |
| Private car |  | 5667 | 55.6 (54.6-56.5) |
| On foot |  | 5863 | 57.5 (56.6-58.4) |
| Others |  | 18453 | 18.2 (17.4-18.9) |

| **Table S4. Residents’ KAP scores towards the public health interventions implemented nationwide** | | |
| --- | --- | --- |
|  | Scores (Mean±SD) | *P* value |
| **Age, years** |  |  |
| ≤20 | 9.9±1.7 |  |
| 21-40 | 10.4±1.6 | <0.0001 |
| 41-60 | 10.6±1.6 | <0.0001 |
| ≥61 | 11.1±1.3 | <0.0001 |
| **Education status** |  |  |
| No formal education/Primary | 9.8±2.2 |  |
| Junior | 10.2±1.8 | 0.139 |
| Senior | 10.4±1.6 | 0.013 |
| College/University | 10.5±1.5 | 0.002 |
| Graduate or above | 10.3±1.8 | 0.061 |
| **Marital status** |  |  |
| Single | 10.3±1.6 |  |
| Married | 10.5±1.6 | <0.0001 |
| Divorced | 9.9±1.9 | 0.035 |
| Widowed | 8.3±2.9 | 0.001 |
| **Family members living together (No.)** |  |  |
| Single | 10.2±1.8 |  |
| 1 | 10.3±1.6 | 0.999 |
| 2 | 10.3±1.7 | 0.611 |
| 3-4 | 10.4±1.6 | 0.031 |
| ≥5 | 10.5±1.5 | 0.007 |
| **Family incomes (rmb per year)** |  |  |
| <50,000 | 10.3±1.7 |  |
| 50,000-120,000 | 10.5±1.5 | <0.001 |
| 130,000-170,000 | 10.4±1.6 | <0.01 |
| 180,000-250,000 | 10.4±1.7 | 0.023 |
| >250,000 | 10.4±1.9 | 0.311 |
| **Current status affected by COVID-19** |  |  |
| Diagnosed, and cured | 8.3±2.1 |  |
| Diagnosed, and under treatment | 7.5±1.9 | 0.581 |
| Suspected, and quarantined | 8.6±2.2 | 0.962 |
| Home-based quarantine | 9.2±2.1 | 0.089 |
| Confirmed healthy after quarantine | 10.2±1.7 | <0.0001 |
| None of above | 10.5±1.5 | <0.0001 |
| **Appearance of clinical symptoms in previous 14 days*** | |  |
| No | 10.5±1.5 |  |
| Yes | 9.7±2.0 | <0.0001 |

*referred to symptoms of fever, cough, expectoration, diarrhea, weak, headache, runny nose, rhinobyon,

- [sore](javascript:;) [throat](javascript:;)
